# MicroPredict: predicting species-level taxonomic abundance of whole-shotgun metagenomic data using only 16S amplicon sequencing data

**DOI:** 10.1007/s13258-024-01514-w

**Published:** 2024-05-03

**Authors:** Chloe Soohyun Jang, Hakin Kim, Donghyun Kim, Buhm Han

**Affiliations:** 1 Department of Biomedical Sciences, Seoul National University College of Medicine, Seoul, South Korea; 2 Interdisciplinary Program for Bioengineering, Seoul National University, Seoul, South Korea

**Keywords:** Microbiome, WGS sequencing, 16S rRNA sequencing, Prediction

## Abstract

**Background:**

The importance of the human microbiome in the analysis of various diseases is emerging. The two main methods used to profile the human microbiome are 16S rRNA gene sequencing (16S sequencing) and whole-genome shotgun sequencing (WGS). Owing to the full coverage of the genome in sequencing, WGS has multiple advantages over 16S sequencing, including higher taxonomic profiling resolution at the species-level and functional profiling analysis. However, 16S sequencing remains widely used because of its relatively low cost. Although WGS is the standard method for obtaining accurate species-level data, we found that 16S sequencing data contained rich information to predict high-resolution species-level abundances with reasonable accuracy.

**Objective:**

In this study, we proposed MicroPredict, a method for accurately predicting WGS-comparable species-level abundance data using 16S taxonomic profile data.

**Methods:**

We employed a mixed model using two key strategies: (1) modeling both sample- and species-specific information for predicting WGS abundances, and (2) accounting for the possible correlations among different species.

**Results:**

We found that MicroPredict outperformed the other machine learning methods.

**Conclusion:**

We expect that our approach will help researchers accurately approximate the species-level abundances of microbiome profiles in datasets for which only cost-effective 16S sequencing has been applied.

**Supplementary Information:**

The online version contains supplementary material available at 10.1007/s13258-024-01514-w.

## Introduction

Research on the human microbiome has increased tremendously in recent years (Berg et al. 2020). Many microbiome studies have found changes in the gut microbial composition in various diseases, such as cancer (Bultman 2014; Wirbel et al. 2019), obesity (Turnbaugh et al. 2006; Tilg and Kaser 2011), inflammatory bowel disease (IBD) ((IIBDGC) et al. 2012), Crohn’s disease (CD) (Gevers et al. 2014; Wright et al. 2015), type 2 diabetes (T2D) (Larsen et al. 2010; Qin et al. 2012), rheumatoid arthritis (RA) (Scher and Abramson 2011), and many other complex metabolic and autoimmune diseases (Mazidi et al. 2016; Duvallet et al. 2017). Since the launch of the Human Microbiome Project (HMP), many statistical methods and computational tools for analyzing microbiome data have been developed to understand the connection between disease and the human microbiome (Turnbaugh et al. 2007; McIntyre et al. 2017; Piro et al. 2017; Sczyrba et al. 2017).

In microbial studies, it is essential to assess the profiles of human gut microbiota in the collected samples. Two methods are widely used for taxonomic classification and abundance estimation: 16S rRNA amplicon sequencing (16S sequencing) and whole shotgun metagenomic sequencing (WGS). The 16S gene sequence is a short region (approximately 1,500 bp) that is well conserved in bacteria and is highly variable among species. Recently, WGS has emerged with technological advances in next-generation sequencing. Because WGS covers the entire genome, it offers high-resolution taxonomic profiling and additional functional profiling. In particular, WGS provides comprehensive and accurate abundance information at the species- level.

However, 16S sequencing and WGS have several drawbacks. Although 16S sequencing is still widely used owing to its low cost, one drawback is that the identification of abundances is mostly focused on the genus level rather than on the species-level. Another drawback is the possible bias in technology, and recent studies have demonstrated that 16S sequencing is biased and imprecise (Louca et al. 2018; Park et al. 2021). This is because of several factors, including the limited representation of microbial diversity in the 16S rRNA gene, the relative abundance of microbes in a sample, and the accuracy of reference databases (although the latter also affects WGS). A drawback of WGS is its high cost. Although sequencing costs have recently decreased, WGS remains significantly more expensive than 16S sequencing. Moreover, computationally extensive processing steps are required to investigate taxonomic annotations because of the large scale of complete genome sequences. Therefore, 16S sequencing remains more widely used than WGS.

Here, we propose a computational method called MicroPredict. Our method predicts species-level abundances that can only be obtained through WGS using 16S sequencing data for the human gut microbiome. Our goal was to predict unknown species-level WGS information while correcting for the possible bias present in the 16S sequencing data. For this purpose, we used two strategies. First, we modeled both sample- and species-specific information to predict WGS abundance. Second, we accounted for possible correlations among the different species. To this end, we built and utilized a mixed model. We subsequently evaluated the performance of our method by comparing it with various machine learning approaches and confirmed that our method outperforms other approaches. We expect that our method will help researchers identify high-resolution species-level taxon abundance without high sequencing costs, computationally intensive data processing, and large storage.

## Materials and methods

### Dataset

To train and test our prediction model, we used human gut microbiome data, for which both 16S sequencing and WGS platforms were applied to the same sample. Through an extensive search, we identified three published datasets (Winglee et al. 2017; Laudadio et al. 2018; Peterson et al. 2021). A brief description of each cohort is provided in Table 1. Below, we explain how we processed the raw data from these three cohort datasets.

**Table 1:** Summary of the three cohort datasets used for MicroPredict training and test

Cohort	Reference	Sequencing platform	Metadata	N	post-QC N
RESONANCE cohort	Peterson, 2021	16S, WGS	C(child)/M(mother) Timepoint (9)	648	405
Urban & Rural (URC) cohort	Winglee, 2017	16S, WGS	R(rural)/U(urban) Timepoint (2)	40	40
Crohn Disease (CD) cohort	Laudadio, 2018	16S, WGS	C(CD)/H(Healthy)	6	6

### Resonance cohort

In the RESONANCE cohort, there were 648 individuals, including human stool sample data from 348 children and 300 mothers (Peterson et al. 2021). Sequencing of the V4–V5 region of the 16S rRNA gene was performed using SRA (NCBI BioProject PRJNA695570), yielding raw sequencing data (.fastq). For more information about the cohort, please refer to the original manuscript.

### Urban and rural Chinese (URC) cohort

In the URC cohort, there were 40 samples of 20 urban and 20 rural people from southern China (Winglee et al. 2017). Sequencing was performed on the V4 hypervariable region of the 16S RNA. Raw sequencing data (.fastq) were obtained using SRA (accession number: SRP114403). For more information about the cohort, please refer to the original manuscript.

### Crohn disease (CD) cohort

In the CD cohort, six different stool samples (three healthy controls and three CD samples) were collected (Laudadio et al. 2018). We used these data as additional independent test data to evaluate the prediction methods because they were too small to train the model. Sequencing of the V4–V5 region of the 16S RNA gene was performed using SRA (NCBI BioProject PRJNA349463), yielding raw sequencing data (.fastq). For more information about the cohort, please refer to the original manuscript.

### Multi-cohort

The RESONANCE and URC cohorts were combined to create a merged cohort. We referred to this cohort as the “Multi-cohort.” To build the final prediction model for users, this Multi-cohort model was used to maximize the number of samples used for model building.

### Taxonomic assignment and quality control (QC)

To profile the microbial communities, we processed the 16S sequencing data using QIIME2 (Quantitative Insights into Microbial Ecology 2 - q2cli version 2021.11.0) with the dada2 plugin (Callahan et al. 2016; Bolyen et al. 2019). We used version 138 of the SILVA database to classify the taxonomy of the reads (Quast et al. 2013). We then filtered low-quality bases and artifacts (barcodes, adapters, and chimeras) and profiled shotgun sequencing data via KneadData v0.10.0 (https://bitbucket.org/biobakery/kneaddata) and MetaPhlAn (version 3.0; 20 Mar 2020 with mpa v30 ChocoPhlAn database) (Beghini et al. 2021). KneadData uses trimmomatic v.0.39 (Alneberg et al. 2014) and Bowtie 2 version 2.4.4 (Langmead and Salzberg 2012) for trimming low-quality reads and aligning sequencing reads to the reference. There are eight hierarchy levels in taxonomic classification; however, we focused only on the lowest taxonomic rank (e.g., species-level) of the taxonomic abundance profiling of metagenomic datasets.

In the RESONANCE cohort, we used all data, including those from adults, to maximize the sample size. As this was a longitudinal study, there were multiple (nine) time points. The earliest time point was selected for each sample. Additionally, we excluded samples that were not paired on either sequencing platform. The data comprised 405 participants: 337 children and 68 mothers (Table 1). We used the child/mother indicator and time point as covariates to control for sampling bias and adjust for significant differences in microbiome composition across conditions. The taxonomic assignment of this cohort resulted in 751 species. A total of 40 samples from the URC cohort were used. Taxonomic assignment resulted in the abundance data for 285 species at the species-level. We used all six samples from the CD cohort. Taxonomic assignment resulted in abundance data for 170 species at the species-level after processing the raw data.

After taxonomic assignment and QC, the taxa were divided into three groups: taxa detected by both platforms, the WGS platform only (WGS only), or the 16S sequencing platform only (16S sequencing only). Table 2 lists the number of species in each group. As expected, WGS was more sensitive for detecting taxa at the species-level compared to the 16S sequencing platform. This contrasts with the results at the genus level, where more taxa were detected by 16S sequencing (Supplementary Table S1).

**Table 2:** Summary of the number of the species for each platform in the three cohorts

Dataset	Both platforms	WGS only	16S sequencing only	Sum
RESONANCE cohort	128	428	195	751
Urban & Rural (URC) cohort	31	236	18	285
Crohn Disease (CD) cohort	32	120	18	170

### Data normalization

Data normalization was required because QIIME2 for 16S sequencing yielded absolute abundances, whereas MetaPhlAn3 for WGS provided relative abundances. To match the units of the two methods, we converted the absolute abundances of the 16S sequencing data to the relative abundances using the QIIME2 “feature-table relative-frequency” function.

After transforming all abundances into relative abundances, we normalized the samples by library size (i.e., total counts). After dividing by the total counts, we multiplied by 10^6^ and applied the log10 transformation to the count values. To avoid infinite values, we added 1 to the values before the logarithmic transformation. This normalization helped us apply our linear mixed model because the original data were likely from count distributions (Poisson or negative binomial) rather than from normal distributions (Dobson and Barnett 2018).

### MicroPredict

**Fig. 1:**
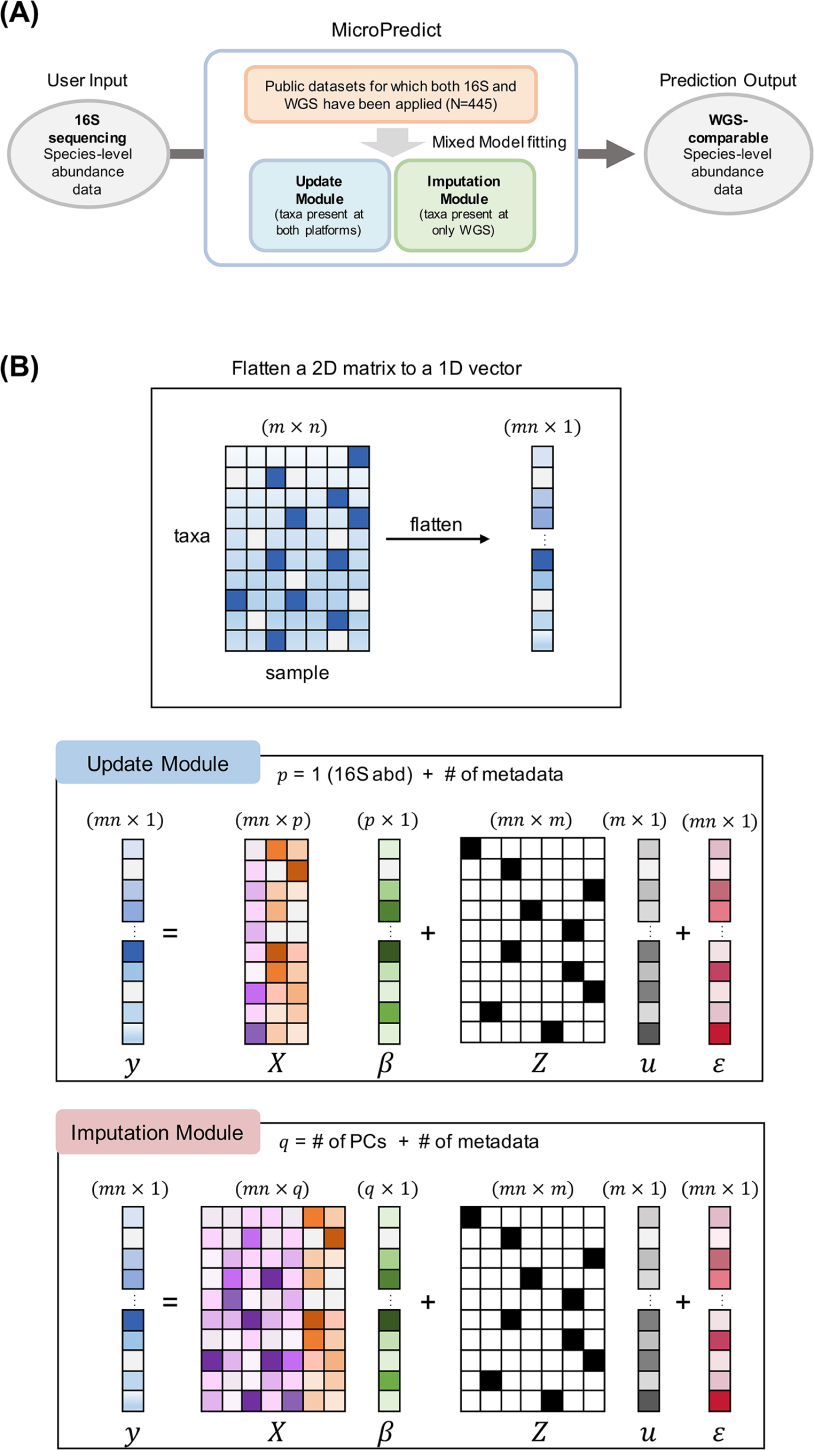
Overview of microPredict. **A** A schematic diagram of MicroPredict. This flowchart illustrates the process of predicting WGS-comparable species-level abundance data from 16S sequencing data using MicroPredict. The user inputs 16S sequencing species-level abundance data. Two modules, the update module and imputation module, are employed for taxa present at both platforms and taxa present at only WGS, respectively. These modules utilize mixed model fitting using public datasets (*N* = 445). The final output is the WGS-comparable species-level abundance data. **B** Visualization of a mixed model of MicroPredict. $y$ is the 1D vector from the flattened WGS abundance 2D matrix. Each module’s model formula is described (with dimensions). The only difference between the update module and imputation module is the fixed effects data matrix ($X$). The update module’s data matrix consists of 16S sequencing abundance and metadata, whereas the imputation module’s data matrix consists of 10 PCs and metadata

We developed MicroPredict, a statistical method that predicts the taxonomic profiles of species-level abundance data using 16S sequencing abundance data. Figure 1A presents an overview of the proposed model, MicroPredict. In this model, we assumed that users had only low-cost 16S sequencing data and aimed to predict the species-level abundance data that would be obtainable using WGS technology. Hence, users can provide a count matrix from the 16S sequencing data as input.

MicroPredict consists of two modules: an update module and an imputation module (Fig. 1A). We first applied the update module to remove possible 16S-specific biases for species present in both platforms. We then applied an imputation module to impute species that were absent in 16S but present in WGS.

### Update module

The update module was designed to predict the taxa detected by both 16S sequencing and WGS. Although these taxa were detected by both platforms, the estimated abundance of these taxa was usually different. This may be because the total number of detected taxa (common denominator) was different or because each platform had its own bias. Here, we assumed that WGS is the gold standard and chose to update the abundances from 16S sequencing to the values estimated by WGS. To this end, we constructed the following linear mixed model:

1 $$y\, = \,X\beta \, + \,Zu\, + \, \epsilon \,$$

$u \sim MVN(0,\sum ),\varepsilon \sim MVN(0,{\sigma ^2}{I_{nm}}),n$ is the number of samples, and *m* is the number of taxa exclusively observed in the WGS platform. $y\in {R}^{nm\times 1}$ is a vector of WGS abundance, and $X\in {R}^{nm\times p}$ is a design matrix consisting of $p$ variables ($p=3$:16S sequencing abundance data, binary metadata, and time point). The metadata variables for the three cohorts are presented in Table 1.

$\beta \in {R}^{p\times 1}$ denotes fixed effects. $Z\in {R}^{nm\times m}$ is a design matrix of $m$ taxonomic species, and $u\in {R}^{m\times 1}$ denotes random effects modeling species-specific effects. $\varepsilon\in {R}^{nm\times 1}$ is a vector of the residuals (random errors). We assume that these two random variables $u$ and $\epsilon$﻿ follow multivariate normal distributions (MVN), where $\varSigma$ is a covariance matrix for random effects $u$. The unknown parameters of this model are $\beta$, $\varSigma$, and ${\sigma }^{2}.$ A schematic of the mathematical structure and dimensions of the proposed model is shown in Fig. 1B.

The rationale behind this mixed model is as follows. The fixed-effect term ($X\beta$) accounts for the sample (individual)-specific effect. The 16S rRNA abundance values of each sample for the same taxa were used as fixed-effect predictors. The random-effects term ($Zu$) models species-specific effects and cross-species correlations. Specifically, species-specific effects were modeled by equivalent shifts in taxa for the same species across all samples. In addition, microbial taxa share evolutionary information that can be used to generate a phylogenetic tree. For example, taxa that are closer together in a phylogenetic tree are likely to share more common features than those that are further apart. The covariance structure ($\varSigma$) encodes these dependencies in the microbiome as correlations between species. We found that prediction accuracy was maximized only when both sample- and species-specific effects were accounted for (as shown in the Results).

### Imputation module

The imputation module imputes taxa that are detectable only by WGS using 16S data. We used a linear mixed model similar to that applied in the update module. The only difference was the use of 10 principal components (PCs) as the fixed-effect predictor instead of the 16S sequencing abundance data of the corresponding taxa (Fig. 1B). The linear mixed model for the imputation module is expressed as follows:

2 $$y = X\beta \, + \,Zu\, + \, \epsilon$$

where $u \sim MVN(0,\sum ),\varepsilon \sim MVN(0,{\sigma ^2}{I_{nm}})$ and $X\in {R}^{nm\times q}$ is a design matrix consisting of $q$ variables $\left(q=12\right). q$ variables were composed of the top 10 sample-specific PCs calculated from the 16S sequencing taxa abundances, binary metadata variables, and time points. Because the PCs summarized the overall abundance of 16S sequencing, the same 10 PCs were used for all imputed species in each sample. The other terms are the same as those given above.

### Mixed model implementation

To implement and solve the mixed model, we used the lme4 package (Bates et al. 2015), which uses restricted maximum likelihood (REML), the standard approach of the mixed model, to estimate the best-unbiased estimates (BLUEs) and predictors (BLUPs). To avoid the estimation of $\varSigma$ into a singular matrix, lme4 reformulates the random effects $u$ as follows. As the covariance matrix $\varSigma$ must be symmetric and positive semi-definite, it can be Cholesky decomposed as ${\Sigma }={{\sigma }^{2}\varLambda }_{\theta }{\varLambda }_{\theta }^{{\prime }}$, where ${\varLambda }_{\theta }\in {R}^{m\times m}$ is a lower triangular matrix. For the computation stability and efficiency, $u$is decomposed such that $u={\varLambda }_{\theta }G$, where $G\in {R}^{m\times 1}$is a spherical random effect and $\theta$ is a vector of parameters consisting of lower triangular elements of a matrix ${\varLambda }_{\theta }$. Equations (1) and (2) can then be reformulated as follows:

3 $$y = X\beta \, + \,Z{\Lambda _\theta }G\, + \, \in$$

where $$G \sim MVN(0,{\sigma ^2}{I_m}){\rm{ }}and\,\varepsilon \sim MVN(0,{\sigma ^2}{I_{nm}})$$.

### Accuracy evaluation

We compared MicroPredict with three methods: linear regression (LR), AutoEncoder (AE), and Convolutional Neural Network (CNN)-AE. As our method is, to the best of our knowledge, the first approach to predict WGS-comparable species-level abundances using 16S data, we had no choice but to compare our MicroPredict with general machine learning methods. The predicted values obtained from these models were compared with the actual abundance values obtained from WGS.

We used two key evaluation metrics: the Pearson correlation coefficient and the root mean square error (RMSE). The Pearson correlation value, ranging between − 1 and 1, measures the linear relationship between the predicted values from each method and the corresponding WGS values. Values closer to 1 indicate stronger positive associations and better agreement. We also employed the RMSE as a metric to assess the absolute goodness-of-fit. The RMSE quantifies the overall error between the predicted and WGS values, with lower values indicating better performance. These metrics were calculated for each dataset and sample.

### Competing methods

#### Standard linear regression

A simple alternative to the same prediction task is the linear regression (LR model, which assumes linearity in the relationship between the dependent variable $(y)$ and the independent variable $(X)$. Our LR model was composed of two modules, similar to MicroPredict. Instead of both random and fixed effects, this model contained only fixed effects. The model can be expressed as follows:

$$y = X\beta \, + \epsilon$$

In the update module, $X\in {R}^{nm\times p}$ is the design matrix with $p$ variables which consist of 16S sequencing taxa abundances, binary metadata variable, and time point. $\beta \in {R}^{p\times 1}$ indicates fixed effects, and $\epsilon$ indicates random errors. In the imputation module, $X\in {R}^{nm\times q}$ is the design matrix with $q$ variables which consist of 10 PCs, binary metadata variable, and time point. $\beta \in {R}^{q\times 1}$ indicates fixed effects, and $\epsilon$ indicates random errors.

### Autoencoder

An autoencoder (AE) is an unsupervised learning technique that uses a neural network consisting of two subnetworks: an encoder and a decoder. The inputs and outputs of the AE are identical. The common goal of the AE is to recover the input data after compressing and adding random noise to the input data. AE can also be used to impute missing data in the input. We hypothesized that the hidden layer could learn the important features of species-level abundance data from WGS and during the training process. Unlike previous models (MicroPredict and LR), the two deep learning models (AE and CNN-AE) were composed of a single module.

There is one hidden layer of the encoder part and one hidden layer of the decoder part before each output layer. All outputs were nonlinearly transformed using a rectified linear unit (ReLU) activation function. We used the mean squared error (MSE) for the loss function and the root mean squared propagation (RMSprop) for the optimizer. For hyperparameters, we used 1e-03 for the learning rate and 20 for the epoch and batch sizes.

During the training process, we trained the AE on merged WGS and 16S sequencing abundance data. In other words, we trained the model using both input (16S) and output (WGS) data. For the intersection group, we trained using species-level abundance data from WGS.

We used only 16S sequencing abundance data for prediction. Thus, we provided 16S sequencing data to the AE while zero-filling in the WGS-only data. The AE was run, the 16S sequencing data in the intersection group were updated to new values (similar to the update module of MicroPredict), and the missing WGS species-level abundance data were filled (similar to the imputation module of MicroPredict). We used the WGS data in the AE output as the prediction result.

### CNN-autoencoder

To improve the performance of the AE, we combined a 1D convolutional layer and a max-pooling layer in the encoder. The overall workflow of the CNN-Autoencoder (CNN-AE) was the same as that of the AE. We used the same loss function as the AE and Adaptive Moment Estimation (Adam) as the optimizer, with the learning rate set to 1e-03. For hyperparameters, we set the epoch to 50 and chose a filter and batch size of 256.

## Results

### Prediction benchmark on two single-study datasets

We benchmarked the prediction accuracy of four methods (MicroPredict, LR, AE, and CNN-AE) on two cohort datasets (RESONANCE and URC) (see Methods). For each cohort, we created a cohort-specific model using each of the four methods. We evaluated the performance of these methods by splitting the data into training and test sets in an 80:20 ratio for each cohort. To compare the accuracy of our method, MicroPredict, with the three other methods, we calculated the Pearson correlation and RMSE between the predicted abundances and true WGS abundances in both the training and test sets. These accuracy metrics were calculated for all samples and species.

The results in Tables 3 and 4 show that MicroPredict consistently outperformed all competing methods across the RESONANCE and URC cohorts. For example, from a correlation perspective, MicroPredict achieved 66.5% accuracy on the RESONANCE cohort test dataset, whereas the second-best method (CNN-AE) achieved only 58.2% accuracy. In addition, MicroPredict demonstrated superior performance compared to the AE and CNN-AE models across various parameters, including different numbers of filters and layers, as detailed in Supplementary Table S2.

**Table 3:** Summary of the number of the species for each platform in the three cohorts

Training accuracy	RESONANCE Cohort	URC Cohort	Multi-Cohort
LR	0.208	0.090	0.198
AE	0.490	0.464	0.463
CNN-AE	0.603	0.472	0.597
MicroPredict	0.659	0.655	0.645
Test Accuracy	RESONANCE Cohort	URC Cohort	Multi-Cohort
LR	0.204	0.091	0.165
AE	0.511	0.534	0.443
CNN-AE	0.582	0.542	0.569
MicroPredict	0.665	0.639	0.638

**Table 4:** Summary of the number of the species for each platform in the three cohorts

Training accuracy	RESONANCE Cohort	URC Cohort	Multi-Cohort
LR	1.820	1.094	1.681
AE	1.804	0.994	1.655
CNN-AE	1.693	0.988	1.544
MicroPredict	1.406	0.836	1.331
Test Accuracy	RESONANCE Cohort	URC Cohort	Multi-Cohort
LR	1.827	1.109	1.683
AE	1.813	0.969	1.638
CNN-AE	1.708	0.965	1.536
MicroPredict	1.398	0.855	1.339

**Fig. 2:**
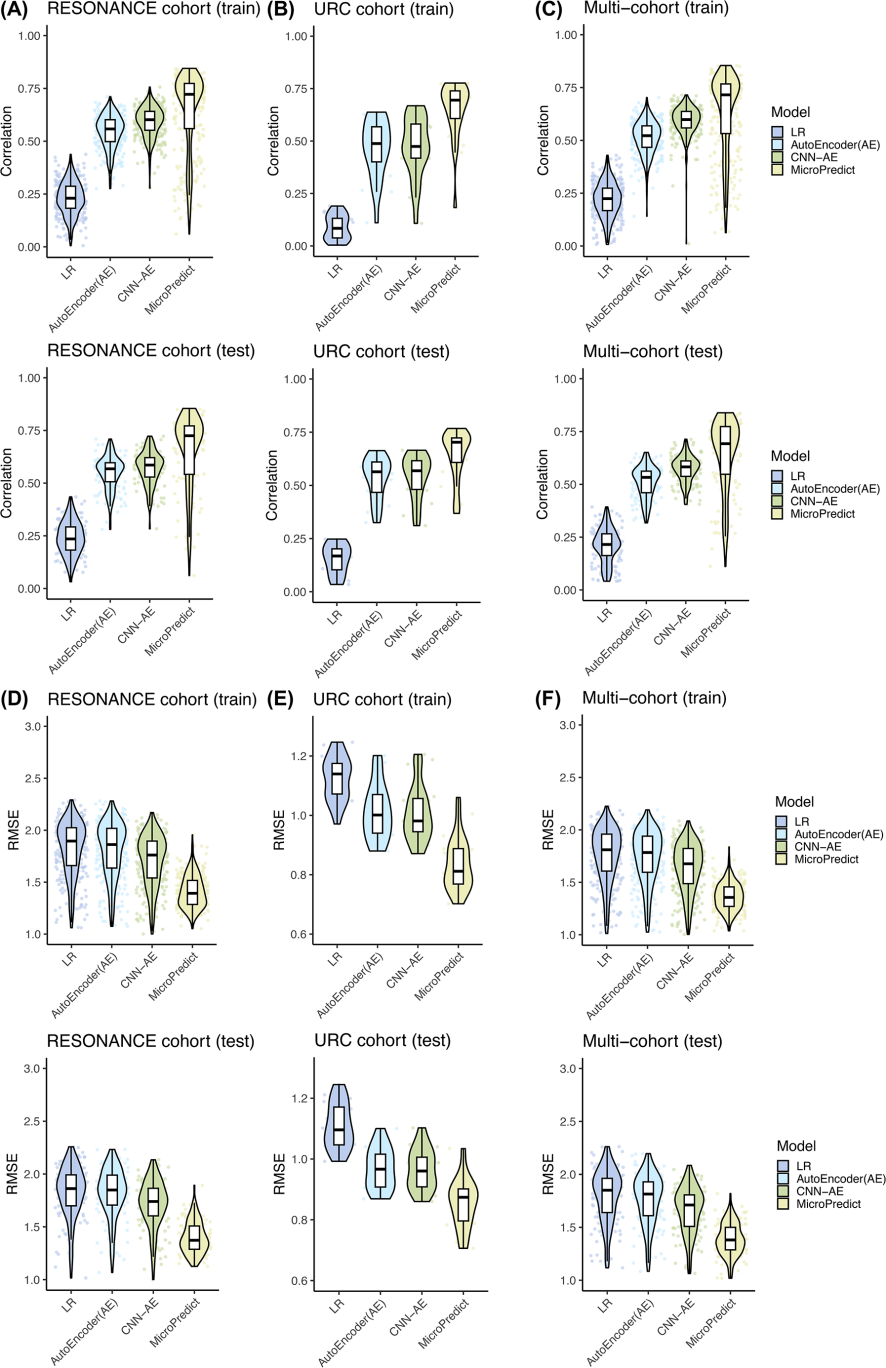
Performance of all four competing models (LR: linear regression, AE: AutoEncoder, CNN-AE: Convolutional Neural Network-AutoEncoder, and MicroPredict) in sample-specific correlation (**A, B, C**) and RMSE (**D, E, F**) for the RESONANCE cohort, URC cohort, and Multi-cohort. Each dot represents a sample, and the performance for both the training and test sets is presented

Next, we calculated the correlation coefficients and RMSE for each sample. Figure 2A, B, D, and 2E show the accuracies of the different methods for all cohorts. We observed that LR had the lowest performance, followed by AE, CNN-AE, and MicroPredict, for all cohorts. Overall, our MicroPredict model achieved superior performance compared to the alternative methods on both the training and test datasets.

To investigate the differences in performance between individual modules (update and imputation modules), we evaluated the performance of each module. The results are summarized in Supplementary Tables S3 and S4. MicroPredict outperformed the other methods on both tasks. Other methods showed significantly different accuracies between the two tasks (particularly LR), but MicroPredict showed high accuracy for both modules. For example, the imputation module achieved an accuracy of 65.6% and the update module achieved an accuracy of 69.2% in the RESONANCE cohort in terms of Pearson correlation.

### Prediction benchmark on multi-cohort model

We made a Multi-cohort model by combining samples from two cohorts (RESONANCE and URC; total *N* = 445) and evaluated the performance of the methods in this merged cohort. Regarding taxonomic composition, this large dataset contained 764 species, consisting of 474 species detected by WGS only, 195 species detected by 16S sequencing only, and 128 species detected by both platforms. Among the 764 species, 33 were assigned to discordant modules in the two cohorts. In other words, 33 species were detected by both platforms in the RESONANCE cohort and were assigned to the update module, whereas they were detected by WGS only in the URC cohort and were assigned to the imputation module. We evaluated 33 intersection species by averaging the two modules after running both modules.

We evaluated the Multi-cohort model by treating it as a single cohort and using the same 80:20 training-test-split as the single-cohort evaluation. The accuracy of the prediction was assessed by calculating the Pearson correlation coefficient and RMSE between the predicted and WGS abundances. When we compared MicroPredict to competing methods in this Multi-cohort (Fig. 2C and F; Tables 3 and 4), MicroPredict consistently achieved the highest performance. MicroPredict achieved the highest Pearson correlation (0.638) and the lowest test RMSE (1.339), representing a 12.13% improvement in correlation and a 12.83% reduction in error over the second-best CNN-AE model.

### Independent evaluation on CD cohort data

To verify how well our proposed MicroPredict method works for a completely independent test set, we used the CD cohort data as a test dataset. We evaluated three different training datasets (RESONANCE cohort, URC cohort, and Multi-cohort). For each training dataset, we used only the overlapping species in the CD cohort for evaluation (Supplementary Table S5). Similar to the previous analysis, we benchmarked MicroPredict, LR, AE, and CNN-AE.

As expected, the performance of all methods decreased in this independent evaluation compared with the cross-validation scheme (Supplementary Tables S6 and S7). This indicated the presence of heterogeneity among the datasets and the challenge of generalizing the model to independent cohorts. Nevertheless, we observed that MicroPredict remained the best-performing model in all tested cases, except for one case; AE slightly outperformed MicroPredict by 0.001 in terms of the correlation for the URC-trained model (Supplementary Table S6).

Different training datasets showed different performances. In particular, the prediction accuracy of all the methods was better when using the RESONANCE cohort than when using the URC cohort as the training model. This may be due to the relatively smaller sample size of URC compared to that of RESONANCE, as well as the small number of overlapping species between the URC and CD cohorts. These results highlight the importance of using appropriate training data with a sufficient sample size for our prediction model for a wide range of applications.

#### Cross-cohort evaluation

Each cohort model (RESONANCE and URC) was validated using a cross-cohort scheme. In other words, we trained the prediction model using one cohort and another as the test dataset. We extracted intersecting species from the two cohorts and evaluated the models for the species found in both cohorts. Species information for the intersection is shown in Supplementary Table S8.

Supplementary Tables S9 and S10 present the results of the cross-cohort evaluation. As expected, compared to the within-dataset benchmark, the performance of all methods declined owing to the heterogeneity between cohorts. Nevertheless, MicroPredict achieved the highest accuracy in both directions of cross-cohort validation compared with the competing methods. For example, the Pearson correlation of MicroPredict was 0.514 in the RESONANCE-to-URC direction and 0.499 in the URC-to-RESONANCE direction, whereas the second-best AE method achieved a Pearson correlation of 0.285 and 0.479, respectively.

### Dissecting model components: species-specific and sample-specific effects

MicroPredict uses a mixed model for both the update and imputation modules. Within each module, there are several mixed-model components that model unique information. The fixed-effects term includes the covariance term (metadata such as group information and time points), 16S sequencing abundance data, and PCs that correspond to sample-specific effects. The random effects term is for modeling species-specific effects and cross-species correlations. If we name these components as COV, 16S, PC, and RE, then the MicroPredict update module can be considered COV + 16S + RE, and the MicroPredict imputation module can be considered COV + PC + RE.

We conducted a detailed investigation into the contribution of each component to the overall predictive accuracy of MicroPredict. Therefore, we evaluated the performance of our method after removing each component to assess its individual impact.

**Fig. 3:**
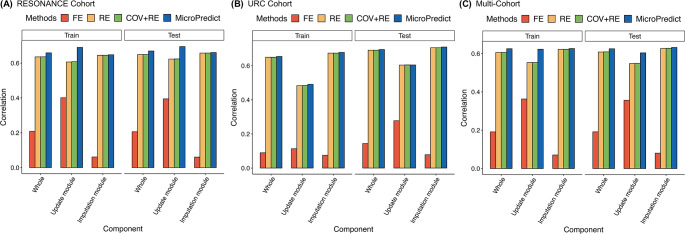
Investigation of the individual effects of each model component on the performance (correlation) of the model for each cohort. The bar plot showcases the performance results of the four submethods: fixed effect (FE), random effect (RE), covariate-only fixed effect with random effect (COV + RE), and MicroPredict. The effects are further divided into three categories: whole, update module, and imputation module. The performance is evaluated using the training and test datasets. **A** The RESONANCE cohort, **B** the URC cohort, and **C** the Multi-cohort

To assess the effects of sample-specific information on MicroPredict’s prediction model, we excluded the 16S sequencing abundance data from the update module and the PCs from the imputation module and obtained a modified mixed-effects model (designated as COV + RE). The evaluation of this modified model demonstrated a slight reduction in imputation accuracy compared to the full model in the RESONANCE cohort and Multi-cohort (Fig. 3). For example, in terms of the Pearson correlation, the performance of the COV + RE models decreased by approximately 3% compared to that of the full models in both cohorts. This suggests that sample-specific information from the PCs positively influenced the predictive accuracy of MicroPredict, albeit to a small extent.

Next, to assess the effects of the covariance term (metadata, such as group information and time point), we excluded the covariance term from the model above and obtained a random effects-only model (RE). We observed that its performance was similar to that of the previous COV + RE model (Fig. 3). For example, in terms of the Pearson correlation, the performance of the COV + RE models decreased by less than 0.3% compared with the RE models in both cohorts. This finding indicates that variables related to groups or time points had a negligible impact on the prediction, at least for these datasets.

Finally, to assess the effects of species-specific information represented in the correlation between species, we built an FE-only model after excluding the random effects term (which we designated as FE). This model is equivalent to the LR model that we used as a comparative model for the performance evaluation of MicroPredict. As shown in Fig. 2, the FE showed the lowest accuracy for all cohorts. This suggests that the correlation between species (random effects term) plays a vital role in predicting species abundance.

In summary, the full model, MicroPredict, consistently outperformed several modified models in which some components were removed. The results of these analyses highlight the importance of the combined effects of several components to achieve a high level of predictive accuracy in the MicroPredict model.

#### Discussion and conclusion

In this study, we developed a method to predict WGS-comparable species-level taxonomic abundance using cost-effective 16S sequencing data as input. Our mixed model effectively incorporated sample-specific, species-specific, and interspecies correlations to obtain high-resolution species-level abundance data.

Although MicroPredict outperforms the existing methods, we acknowledge several limitations. First, our method is constrained to predict the abundances of microbial taxonomic classifications present in the training sets. A single training dataset cannot cover all existing gut microorganisms, given the variations influenced by factors such as host diet and disease status. Thus, a large training dataset that includes as many individuals as possible is essential for building a robust model. However, to be used as a training set for our prediction model, a sample dataset must be processed using both the 16S sequencing and WGS platforms. We found that many public datasets were processed using only 16S sequencing, which made it difficult to build a large training dataset. As more studies are being conducted with both technologies in the future, we expect that the prediction accuracy will increase. Furthermore, we expect that our method will be applicable to different contexts of microbiome studies other than the human gut microbiome when a suitable training dataset is available.

Second, the prediction performance of MicroPredict decreased when there was heterogeneity between the training and test data. This was evident in our analysis, in which the cross-cohort prediction accuracy was lower than that of the within-cohort benchmark. This is an inherent limitation of all the existing prediction models. We expect that this limitation can be ameliorated if we can collect a large training dataset that includes a large number of different conditions.

Third, our method can only predict WGS-comparable abundance data at the species-level but not actual WGS sequence data. One clear advantage of using WGS data is the ability to analyze sequence information. For example, HUMAnN 2.0 (Franzosa et al. 2018) uses the sequence data for functional annotation by referencing it with the database in the functional profiling process. As our method can only predict abundance data, it does not allow for direct functional downstream analysis, such as gene or pathway profiling, which would have been possible with actual WGS data.

Fourth, although the WGS platform is clearly more sensitive for detecting high-resolution taxa at the species-level than the 16S sequencing platform, WGS may still not be perfect. In our analysis, a few species-level taxa were detected by 16S sequencing and not by WGS. The reason for this may be that the WGS reference database is relatively new and is still growing, whereas the 16S reference database is well curated and established. Hence, for some species, 16S sequencing may be more sensitive than WGS at the current stage. However, it remains unclear whether these 16S-only-detected species are correctly annotated. 16S sequencing is also known for biased results. It is possible that these species were incorrectly annotated using 16S due to limited information. We expect that as the WGS database becomes more updated and stable in the future, WGS will become more accurate, sensitive, and unbiased.

Despite these limitations, MicroPredict allows researchers to obtain high-resolution taxonomic profiling abundance data using only 16S sequencing data. Compared with WGS, 16S sequencing data are more abundant in the research community because of their lower cost and longer history. We expect that by using our method, researchers will be able to extract useful information from a myriad of existing 16S data to understand the role of the gut microbiome in human health and disease.

Our research on the human gut microbiome suggests that our predictive model might extend to other organisms, such as mice. To achieve this, we would need comprehensive datasets utilizing both 16S sequencing and WGS methods for each organism. This approach could expand our research and provide insights into microbial communities across diverse species. We hope that this research can be extended in future studies, either as an additional study or as a follow-up to our current work.

### Electronic supplementary material

Below is the link to the electronic supplementary material.

Supplementary Material 1

## Data Availability

The source code for the application can be found via the following URL: https://github.com/hanlab-SNU/MicroPredict.
